# Supplementary material for: Schistosomiasis messaging in endemic communities: Lessons and implications for interventions from rural Uganda, a rapid ethnographic assessment study
Source: PLoS Negl Trop Dis. 2021 Oct 27;15(10):e0009893. doi: 10.1371/journal.pntd.0009893 (PMC8575311; doi:10.1371/journal.pntd.0009893)
Supplement: S1 Text — (DOCX) [file pntd.0009893.s001.docx]

**Supporting Information: S1 Text: Topic guide for the focus group discussions and in-depth interviews**

## Focus Group Discussion Guides

**Introductory Group Session** (8-10 community leaders)

Introduction

- Introductions
  - researchers and participants introduce themselves
- Explain the study
  - researchers introduce project aims, and aims and methods of WP1
- Consent
  - participants ask any questions they have about the study
  - participants provide written/thumbprint consent to take part in this introductory group session
  - the on-going nature of consent reiterated
- Ground rules
  - Respect for other speakers, no interrupting, confidentiality

The Community

- What kind of work do people in the village do?
  - PROMPT: car washing, subsistence agriculture, market agriculture, fishing
- Where do people access water for
  - Drinking
  - Washing
- Where are the toilet facilities in the village (PROMPT: houses, school, public etc)
  - What do they look like (PROMPT: flush toilet, pit latrine, trench latrine, ventilation improve latrine, other)?
- Where do you think different people go to the toilet?
  - PROMPT: open defecation, latrine, lake
  - PROMPT: mothers, fathers, elderly people, children, fishermen, farmers
  - OPEN DEFECATION PROMPT: where do you think people go?
    - The lake, fields? Close to village? Far from village? Into running water? Away from running water?

Health

- Key health concerns in the community
- What resources do you have to respond to them?
- What resources would help?

Schistosomiasis

- Have you heard of schistosomiasis?
  - What is it? *(leave participants free to describe unprompted)*
  - Where have you learned about schistosomiasis?
    - PROMPT: health centre, community education, children, other
- How can you tell if someone has schisto?
  - PROMPT: different if babies, children, adults?
- How do people catch schisto?
  - PROMPT: different if babies, children, adults?
- Can schisto be treated? How?
  - What treatment is available? To who?
  - Is treatment important? (Why/not?)
  - Barriers to MDA uptake
- What (if anything) do people in your community do to reduce the risk of *getting* schisto?
  - PROMPT: boil/filter washing water before use, stop children swimming in the lake etc
  - Barriers to doing these things? For who in particular?
- What (if anything) do people in your community do to reduce the risk of *transmitting* schisto?
  - PROMPT: avoid defecating in/near streams, avoid defecating in lake etc
  - Barriers to doing these things? For who in particular?

Ending

- If you could change 3 things in your village to reduce schisto transmission, what would they be?
  - PROMPT: material infrastructure, education, everyday practices
  - What are the barriers to those changes?
- Reminder about confidentiality, right to withdraw responses/from study. Thank you.
