# Supplementary material for: Schistosomiasis messaging in endemic communities: Lessons and implications for interventions from rural Uganda, a rapid ethnographic assessment study
Source: PLoS Negl Trop Dis. 2021 Oct 27;15(10):e0009893. doi: 10.1371/journal.pntd.0009893 (PMC8575311; doi:10.1371/journal.pntd.0009893)
Supplement: S1 Table — (DOCX) [file pntd.0009893.s002.docx]

**Supporting Information: S1 Table: Coding framework showing the group and sub-group codes used for data analyses.**

| **Overarching Group Code** | **Sub-Group Code** | **What it Covers** |
| --- | --- | --- |
| **Barriers** | - | The things that make it difficult for people to practice good behaviours in relation to bilharzia transmission |
|  | Cost | Anything to do with money (e.g. cost of water, firewood, difficulty finding work to earn money) |
|  | Distrust | Always about people – ie not trusting individuals (e.g. VHT is trying to kill me with MDA), the government (they’re experimenting on us), white people (we didn’t have bilharzia until white people came) etc. |
|  | General Problems | Things that make life difficult (e.g. lack of rain) |
|  | Lack of access | Broken taps, boreholes, no latrine at home, no public latrine you are able to use (e.g. fishermen cannot use SPINCON latrine) etc. |
|  | Religion | Religion prevents e.g. MDA use |
|  |  |  |
| **Ideas about bilharzia** | - | Anything to do with how people talk about bilharzia |
|  | Sources of information | Where do they get information about bilharzia (does not need to be accurate) e.g. VHT, radio, friends, family |
|  | How you catch bilharzia and transmission | How people think you catch it, how it is transmitted |
|  | How you treat bilharzia | Any ideas about treatment (inc. traditional healers) |
|  | Ekidada | Anything people say about ekidada at all |
|  |  |  |
| **Risk management & solutions** | Desired solutions | What things do people want? Can people answer the question? |
|  | Protective but other motives | Things people do that reduce transmission but are done for other reasons |
|  | Things individuals do to protect themselves | E.g. Wearing gumboots, fetching water from wells, building own latrine. Also if people say they don’t do anything to protect themselves. (Excluding water processing, which was included in a separate sub-group below) |
|  | Things leaders do to protect communities | Write or enforce bylaws around e.g. each household must have a latrine, fine people for open defecation |
|  | Water processing | E.g. boiling, filtering, leaving in the sun |
|  |  |  |
| **Risk to others** | - | What are the things people do that either put others at risk or people think put others at risk? |
|  | Other | Any other risks to others not covered by other child codes |
|  | Open defecation | Any defecation not in a latrine, e.g. on stones, in the lake, in polythene bags |
|  | Toilets as cause of infection | Anything where people talk about toilets as transmitting any disease or general illness |
|  |  |  |
| **Risks to self** | - | Things people do that put themselves at risk or that they perceive as putting themselves at risk |
|  | Bathing | Any water contact (lake water or not) relating to bathing |
|  | Drinking and cooking | Any water contact (lake water or not) relating to drinking and cooking |
|  | Fetching lake/swamp water | Anything at all about fetching water |
|  | Fishing | Anything at all about fishing |
|  | General hygiene | Anything at all about general hygiene |
|  | Swimming | Anything at all about swimming |
|  | Washing vehicles | Anything at all about washing vehicles |
|  | Washing clothes | Anything at all about washing clothes, regardless of water source |
|  |  |  |
| **Clean & dirty** | Latrine coverage | Descriptions of presence/absence of latrines. People talking about the presence or lack of latrines, or public latrines that have been built and become full etc |
|  | Descriptions of landscape | Most likely to be from observations and transect walks – any descriptions of what the communities look like |
|  | Things described as clean or dirty | Anything at all described as ‘clean’, ‘safe’, ‘pure’ etc, or as ‘dirty’, ‘polluting’, ‘unclean’ etc, eg I fetch water in the morning because the water is cleaner before the waves make it dirty |
|  |  |  |
| **Other** | - | Anything else that might be interesting |
|  | Culture | Anything you think is a consequence of Ugandan/Lusoga culture. Anything participants describe as being because of culture, inc. discussion of e.g. fishermen behave that way because they are fishermen |
|  | Greetings | For the sake of completion, code for greetings here. |
|  | Social influence | Anything at all about the ways in which some people influence others, e.g. leaders use their position to encourage behaviour change, or someone reports side effects from MDA to friends and that deters those friends from using MDA |
|  | General health | Anything at all about health that isn’t specifically on bilharzia. If people include bilharzia alongside other health problems in the community, code it here. |
