# Supplementary material for: Schistosomiasis messaging in endemic communities: Lessons and implications for interventions from rural Uganda, a rapid ethnographic assessment study
Source: PLoS Negl Trop Dis. 2021 Oct 27;15(10):e0009893. doi: 10.1371/journal.pntd.0009893 (PMC8575311; doi:10.1371/journal.pntd.0009893)
Supplement: S1 Data — (DOCX) [file pntd.0009893.s003.docx]

**Supporting Information: S1 Data: Raw anonymised data on messaging.**

**Messages about other issues which have worked- talk shows about family planning**

2017.09.05 Bugoto IDI

“previously women were not utilizing family planning methods because of what they used to hear from friends but of recent after having a lot of radio talk shows explaining family planning methods, many women are now utilizing them, so for bilharzia if one person takes drugs and feels bad he will spoil other 10 people that they shouldn’t take praziquantel because it is bad, therefore if bilharzia is also given radio talk shows you never know people will get to know the benefits of praziquantel”

**Officials commercialize Bilharzia messages**

2017.09.05 Bugoto IDI

One of the community guides I was moving with told him that previously they had such radio programs but top officials commercialized bilharzia because some wanted to gain money from it, that before they had Schistosomiasis Control Initiative but in 2007 NTD neglected transmitted diseases came up yet the two had conflicting interests over money.

**Sources of information about Bilharzia**

**Photos used to illustrate bilharzia**

2017.09.06 Bugoto IDI

I asked them whether they have ever seen someone with bilharzia and they said health workers showed them photos and they saw them with swollen stomachs.

**VHT source of information and what they say**

2017.09.12 Bugoto FGD

The Village Health Team sensitize them not to drink that water. They also tell them that if one defecates in the lake when he has bilharzia, any other person who accesses the lake or pond stands high chances of catching bilharzia. They teach them that snails also produce parasites that cause bilharzia.

2017.10.10. Bugoto IDI

I: How about getting to know about bilharzia from health centres?

R: No I have never, I only get such information when VHT’s move to give us tablets.

2017.10.11. Bugoto IDI

R: It is health workers, the VHT’s mobilise us and they sensitise us about any illness they want.

2017.09.12 Bugoto FGD

**Researchers**

R.2: Dr xx would move with her team in the community and whenever the team came around and it used to move from one household to another while sensitizing people

R.2: So the individual who was taking notes would conclude by informing them that bilharzia is parasitic and one catches it when they access infected water. It usually affects people who over stay in water, play in water, spend a lot of time at rice farms, people who drink water un boiled water and she noted that it passes through the hair follicles. While still sensitising, she would conclude by telling them that when the parasite enters someone’s body, it affects the liver and when the liver is perforated, it starts to swell but it’s quite hard for individuals to identify that during the early stages. It usually happens after some time. So, when the stomach starts to swell, that’s when some people start to refer to it as ekidadda. She would still advise the household members to fetch water from the lake as early as 6:00am and thereafter leave it in the sun. She noted that before 10 :00am, the parasite in not active. So, the time they left, people had understood what is bilharzia and how its spread because they endeavoured to sensitise people.

2017.09.12 Bugoto FGD

R.3: The health workers that treat bilharzia are the ones who came and taught us

2017.09.14 Bugoto IDI

I: I was also called at health center II, that’s where those health workers came that taught me about how bilharzia spreads and how you are supposed to prevent yourself from contracting it but they never left behind a strong foundation?

2017.09.12 Bugoto FGD

The VHT’s sensitize them not to drink that water. They also tell them that if one defecates in the lake when he has bilharzia, any other person who accesses the lake or pond stands high chances of catching bilharzia. They teach them that snails also produce parasites that cause bilharzia.

2017.09.12 Bugoto FGD

R.3: There is a team that brought us reference books that had information concerning bilharzia; they were labelled [Male Name commonly used in Central Uganda] and Bilharzia

2017.09.12 Bugoto FGD

**What has worked for Bilharzia sensitisation – schools and other**

R.3: They were distributed at schools in order for the students to read

2017.09.14 Bugoto IDI

R: I heard about it some time back when I was still studying but during that time I didn’t take it serious because in our place- xxxx it wasn’t so common but when I came here, it was a common topic. I also heard from people at the health department. Everybody was talking about bilharzia and even today I have heard it from you

2017.09.14 Bugoto IDI

even before the outbreak of bilharzia we used to get water from the lake and we could drink without boiling but when things worsened and we started falling sick, we were told to stop drinking lake water and we were told to start boiling the water.

I: Who told you?

R: We had health workers who told us, we were also encouraged by community leaders because they wanted their subjects to be healthy because we used to have diarrhoea, vomit, the water had algae. So that water was un drinkable.

2017.09.14 Bugoto IDI

Those concerned with health, Beach Management Unit leaders, they told us it is not good to wash from the lake.

2017.09.14 Bugoto IDI

I: Let us now talk about bilharzia. Have you ever heard about bilharzia?

R: We hear about it on radio and also from VHT’s, we also get to know about bilharzia from our friends with swollen stomachs

R: I learnt from community education, they bring us community education and also give us tablets that we use.

R: We also get the information from children because heath workers go to schools and check them, they ask us to consent for the children to be treated

2017.09.14 Bugoto IDI

R: I learnt from the other health workers I told you about, the ones who came and gave us tablets, they first taught us about it before giving us tablets.

R: The health workers I have talked about went to schools and taught children about bilharzia, they took off their stool and blood for check-up and when children came back, they told me that they were checked bilharzia.

2017.09.15 Bugoto Observation

I approached this young boy being the first person I saw that day in gum boots and asked him why he was in gum boats, he told me that he just wanted to put on gum boots that day, I asked why he wanted and said that he hears on radio that when you step in lake water with gum boats you may not get bilharzia worms

2017.09.14 Bugoto IDI

I: How about getting information about bilharzia from children?

R: The health workers I have talked about went to schools and taught children about bilharzia, they took off their stool and blood for check-up and when children came back, they told me that they were checked bilharzia.

2017.09.15 Bugoto IDI

Have you ever heard of bilharzia?

R: I have heard a lot about it and I have seen pictures of it in those health centers, I have also seen that some one that is suffering from it and I looked keenly at them and the situation they were going through was indeed terrible, they may fatten the legs and the stomach, their respiratory will be bad, the way they walk too, so by the time God calls them, they die a very disturbing death with so much pain, the foot will have grown fat and so shall the stomach.

2017.10.12 Bugoto FGD

P9: Am amongst the people who get tablets in MDA, I got that information when I was going to be treated.

2017.10.12 Bugoto FGD

P4: We know from news, radios, television and news papers also.

2017.09.25 Bugoto IDI

R: We were at school and the other training we received was at the health centre and this was all about bilharzia

2017.10.10 Bugoto IDI

R: I learnt about it long ago because they used to check us bilharzia.

I: From where?

R: At school.

2017.09.25 Bugoto IDI

I: Did you hear anything about bilharzia from health facilities?

R: Eeeh, there are health charts at the health facility showing how you catch it, its effects and what to do in case you caught it.

2017.10.10 Bugoto IDI

R: This bilharzia, I learnt about it from when it had affected some people and an organization came and sensitized us so every time I could see people that have fattened, and they also came and said that if a person has it we call it witchcraft so we could ask you to get traditional medicine, it’s of late that we learnt that bilharzia is a disease and once someone begins to fatten, and the fatten the stomach

2017.10.12 Bugoto FGD

R.3: Some of us hear it from the health workers, they are the ones that tell us about the bilharzia disease and how it is caused

2017.10.11. Bugoto IDI

R: Health workers used to bring at sub counties. They used to invite us on a public radio that on this date you come here. And we could go.

I: Okay.

R: In the film they used to show us that it is a person who has bilharzia who spreads to others by defecating and urinating in the lake, also fishermen with bilharzia defecating in the lake is what causes bilharzia.

I: You told me that you learnt from cinema, when was that?

R: It was in 1956. I was still in xxxx

2017.10.12 Bugoto FGD

R.7: Those health workers that sensitize us about bilharzia normally tell us that there are minimal chances of you getting the bilharzia worm in the water if you fetched water in the morning so you are most likely not to contract bilharzia. That’s why we normally go very early in the morning.

2017.10.12 Bugoto FGD

R.2: There are also posts displayed about bilharzia for those that can read and even these people that come around to sensitise helps us to learn about it

I: You said you get to hear about it from the health center, community education, on the radios. Anyway, which person do you think is at risk of catching bilharzia?

2017.10.12 Bugoto FGD

P9: Am amongst the people who get tablets in MDA, I got that information when I was going to be treated.

2017.10.12 Bugoto FGD

P2: Me to understand the issue of using water guard, I knew from xxxx health centre but some years back that I don’t even remember, health workers came and taught us how we acquire bilharzia from water, they told us to be getting water guard from clinics then put in water so that the parasites go down and then drink the water, they used to tell us not to drink water without putting in water guard.

2017.10.12 Bugoto FGD

P7: We just hear bilharzia and on radio they even make announcements about it, I hear that for bilharzia the stomach swells, some people call it ekidada, the limbs also swell and people say it is elephantiasis, so is that what they call bilharzia? I cannot differentiate.

2017.10.12 Bugoto FGD

P8: Actually for me when I came back from school and told my father that we were given bilharzia tablets, he asked what bilharzia is but I failed to answer him.

2017.10.13 Bugoto FGD

P1: Bilharzia team came in our area and showed us videos of how one catches bilharzia and we saw, someone stands in water and the parasite runs towards you and enter the joints, then you start feeling limb pain without knowing that it is the parasites then it reaches the intestines.

2017.10.13 Bugoto FGD

P12: Even on radios they announce.

I: How about getting information from this health centre?

P2: No. they don’t have time to teach us.

P6: We just see health charts with pictures of those with swollen stomachs, swollen legs, one with small legs but with swollen stomach.

P8: And they tell us that those children suffer from kwashiorkor, and kwashiorkor is in English we don’t understand what it is. health workers tell us it is kwashiorkor and not bilharzia yet for us we know kwashiorkor also makes someone to swell like that.

2017.10.13 Bugoto FGD

P2: They start from 8 years and above, then for adults if you are given and you don’t get any side effect you get to know you are bilharzia free but if you get side effects like diarrhoea, you get to know you have bilharzia, and you really over defecate after swallowing.

2017.10.13. Bugoto IDI

R: It is mostly our wives who attend community education.

2017.10.13. Bugoto IDI

R: People have not yet understood the issue of leaving water in the sun. that announcement is always on the radio about leaving water in the sun for 24 hours before use, for them after fetching water if they are going to bath a baby or cook food they just use the water without doing anything on it.

2017.11.04 Musubi FGD

P7: We always get health workers who check us, they also give us drugs and some people get healed.

2017.11.04 Musubi FGD

P1: Some of those health workers come from Mayuge

P3: They could take samples from people and they could walk in the community together with us the VHT’s.

2017.11.10 Musubi FGD

P10: We have heard on radio and have also watched on television.

2017.11.10 Musubi FGD

P9: Me I got to know about it from this school and I remember they even showed us videos of how bilharzia is caught.

I: When was that?

P9: around 2007.

P3: Around 2008.

2017.12.11 Musubi IDI

I: What does the advert say?

R: The advert goes, where do you defecate? I defecate in the lake, where do you fetch water? I fetch from the lake; eeh you are the ones who have caused bilharzia. *(laughs)*

2017.12.14 Musubi IDI

R: They usually to advise us over the radios to leave water under the sun for 24 hours before use and they also encourage farmers and fishermen to wear gumboots

2017.12.14 Musubi IDI

R: I heard someone on radio saying that bilharzia is ekidada but we got confused because we have people who have suffered from ekidada, there is one we even took to Jinja referral hospital, she was bewitched ekidada when she was pregnant and by the time she delivered…

2017.12.14 Musubi IDI

R: Uuuh, they confused us by saying there is no ekidada that is bewitched. And they said there is ekidada (bilharzia) found in water and lake. We hear on radio, I don’t know whether you also confirm that there is no ekidada that is bewitched but there is ekidada caught through water, I don’t know your view.

2017.12.14 Musubi IDI

R: I just hear people talking about bilharzia but I don’t know it’s meaning. I don’t know that illness.

2017.12.18 Musubi IDI

I: You said that if it is true you catch it from the lake, are you not sure?

R: Am sure because I have seen many being treated but I didn’t know where they catch it from that’s why I said if it is true because I just listen on radio, I have never been taught individually.

2017.12.18 Musubi IDI

R: I have been hearing announcements, health workers also come to this community and tell us about it, they also give us tablets to swallow. And what shows that we have bilharzia when we swallow the tablets we feel bad, after swallowing you find someone lying down and vomiting.

2017.12.18. Musubi IDI

R: VHT (SN) taught us that bilharzia are parasites found in the lake, they make the stomach swell.

2018.02.08 Bwondha FGD

R.2: They sensitize over the radio and even made a song out of it. They say that you can catch bilharzia if you do open defecation

R.3: You can get a swollen stomach but then, you have to boil water

R.5: You ought to drink boiled water

2018.02.12 Bwondha IDI

R: Health workers came here who gave us that information and tablets,

2018.02.16 Bwondha IDI

I: Where did you learn about bilharzia?

R: I learnt from school. health workers used to come and they could tell us to go to school to get tablets.

I: How about getting information through community education?

R: Me I rarely attend community education.

2018.03.10 Bugoto FGD

P9: There is a health chart in the health facility and a book also.

P6: Madam gave us a book that had a child with a swollen stomach.

2018.03.14 Bwondha FGD

I: You talked of radio; what do they talk about when mentioning bilharzia and how do they define it?

R.1: They usually say that people who are close to the lake should prevent themselves against bilharzia because of open defecation

R.9: They say that people do open defecation at the lake/shore yet their faeces contains worms. Remember one may bathe with lake water without boiling, leaving it in the sun or even drink it hence getting bilharzia. On a contrary, even us in this discussion could be among the people who do the open defecation

R.8: They say that if you go to fetch water, you shouldn’t spend a lot of time in the lake because the worm enters through the hair follicles

2018.03.14 Bwondha FGD

P4: I have been in this community and I always ensure that I get to know new things in the community but I have never heard about any community education of bilharzia, they call us to be tested HIV and other things but not to sensitise about bilharzia. And the people who came to teach us about bilharzia came like some ten years back.

P7: I think you are the first person to call us and talk about bilharzia.

2018.06.04 Bugoto FGD

I: You earlier mentioned that they talk about bilharzia over the radios but what do they specifically say or how do they explain what bilharzia is?

R.6: Some of us are not keen to listen

I: You do not do that?

R.4: Some of us are out there just to listen to the wording- bilharzia and we move away

**Comments about Bilharzia that reflect some talking or rumours in the community**

2017.09.25 Bugoto IDI

I: Are you referring to the prevention medicine?

R: Yes, health worker, one usually develops serious diarrhoea and one can say that this medicine was given to him or its some sort of disease they developed to make him die.

2017.09.25 Bugoto IDI

I: What are the barriers to Mass Drug Administration?

R: First of all, its ignorance; there people who say that they got fed up of government drugs since the drugs intend to reduce on people’s life span. Then there those who say big; some say that they are for TASO (HIV treatment)

2017.09.25 Bugoto IDI

I: What are the barriers to MDA?

R: Hmmm, some women say that when you swallow bilharzia tablets you stop giving birth, they say I cannot swallow them, others say that those tablets are for illuminati. Everyone speaks in his or her own way.
