# Supplementary material for: Schistosomiasis messaging in endemic communities: Lessons and implications for interventions from rural Uganda, a rapid ethnographic assessment study
Source: PLoS Negl Trop Dis. 2021 Oct 27;15(10):e0009893. doi: 10.1371/journal.pntd.0009893 (PMC8575311; doi:10.1371/journal.pntd.0009893)
Supplement: Attachment — Submitted filename: Ssali et al. Responses to reviewers.docx [file pntd.0009893.s004.docx]

**RE: Submission (PNTD-D-21-00619) - [EMID: a59725733ffe8c98]**

Dear Prof. Anne Rimoin and Prof. Jaap van Hellemond,

Thank you for taking time to process and obtain reviews for our article ‘Schistosomiasis messaging in endemic communities: lessons and implications for interventions from rural Uganda, a rapid ethnographic assessment study’. We are grateful for the valuable comments received from the reviewers. We have done our best to address them, as described below in bold, taking each point in turn, and we believe that the manuscript is improved by them and hope that you agree it is now suitable for publication in *PLoS Neglected Tropical Diseases*.

Where we refer to page and line numbers, we are referring to the version of the full manuscript with track changes.

Reviewer 1 general comments

Reviewer #1: - The objectives are clearly stated and the study design was appropriate to address the objectives. Three schistosomiasis-endemic communities in Uganda were selected for this study. Data were collected using a rapid ethnographic assessment approach by two research assistants who had spent 6 weeks at each community. The methods include interviews and FGD as well as observations.

A thematic analysis was considered. The study design and utilized methods are not meant to produce quantitative data and statistical results. However, I recommend adding a table or a box for the rapid ethnographic assessment approach of schistosomiasis as an ethnographic field guide for future studies in other endemic communities in Uganda or elsewhere.

**Response: A table has now been added under study design on pages 8-10 to provide a description of the rapid ethnographic assessment components.**

Moreover, more details about the studied communities are required (Refer to Summary and General Comments #3).

**Response: we have added additional details about the studied communities `Study area’ on page 6 and a map as Figure 1.**

In addition, the study justification should be improved including the using of the rapid ethnographic assessment approach. Refer to the comments listed in the "Summary and General Comments".

**Response: we have added the following text on page 5.**

**The results presented here are from a rapid ethnographic assessment undertaken to explore specific reasons why the schistosomiasis burden has remained high in three lakeshore communities, despite long-term MDA. This exploratory method enabled the understandings, priorities, and proposed solutions of community members to be identified and explored. A key theme among the suggestions made by community members was the need for improved messaging, which is the focus of this paper**.

Reviewer 2 General comments

Are the introductory FGDs and the other FGDs different?

**Response: No, the focus of the groups was the same, but the difference was in terms of who attended, the introductory FGD was held with leaders and influential persons in the communities (homogenous in terms of their role within these introductory FGDs) and they included both men and women (heterogeneous in gender in the discussions). This is now clarified in the table and in the third paragraph of the data collection section.**

Did you use the same topic guide for both (introductory and other) FGDs?

**Response: Yes, the same topic guide was used and this is now clarified in the table and the text in the third paragraph of the data collection section.**

Did you use the data from the introductory FGDs to analyze?

**Response: Data from all the FDGs including the leaders (Introductory FGDs) were used in the analyses and this is now clarified in the text in the third paragraph of the data collection section.**

Who/how did you select individuals as the samples of IDI in the communities?

**Response: Thank you for highlighting this omission. We have revised the information provided in the section on the Study population (page 6) and study design (page 7) to explain how individuals were selected.**

Was the data saturated?

**Response: As a rapid ethnographic assessment study design, we did not aim for data saturation. While data saturation is a concept well suited to grounded-theory based theory building, our aims were to generate rich, textured data on understandings of, practices relating to and ideas about solutions around schistosomiasis to help identify best bet solutions to a specific problem in a time-bounded way. We have described our aims, the activities we undertook, and the people we spoke to within this tight rapid ethnographic assessment time frame to provide readers with context. While we did find that by the end of data collection no new themes were emerging, due to the structure of the study data collection and analysis being undertaking discreetly, this cannot be called true data saturation.**

Specific reviewer comments

Reviewer #1: The results are presented in a narrative manner. The narrative is clear and rich in details. However, some information on the respondents or study activities and communities can be provided in a table. Few comments related to results are listed in the "Summary and General Comments".

**Response: While we appreciate this reviewer’s comment, it is the richness that is enabled by a narrative structure that we feel is the key strength of the paper and our findings. We have therefore chosen not to present results in table form in order to communicate consistently to readers the richness, complexity and heterogeneity of the findings and of the lived world. We have however provided a table providing more details on the study activities and the people who participated in them, as well as more information on the communities where the study was undertaken.**

**Reviewer #2**: - Lines 227-231: This part describes the ordinal situation of the study site. It might be in the method part. (I think it is not a result of this study.)

**Response: Thank you for pointing this out, we have now moved this to the methods, Study area, section.**

How many people participated in the FGDs?

**Response: Thank you for highlighting this omission. We included 192 adults and 40 children. This has been added to the Study population section and at the start of the results.**

Could you describe the characteristics of the participants of FGDs and IDI?

**Response: We have now described the participants in the section called Study population.**

Could you show how many codes and categories you created?

**Response: We have now included information on this in the main text in the data management and analysis section and the code book has been added as supplementary material: 6 overarching group codes, and 30 sub-group codes.**

Line 279: It shows “18-30 years FGDs”, however, in the method section, you described the age groups as “18-34 years” and “35+ years”, please confirm it. Line 404 in the discussion section has the same issue. Please check it too.

**Response: Sorry for this error, these have been corrected to 34 years throughout.**

This is just my interest, “watching videos and films” (line 283), do you know where they watched it? -

**Response: We have added to the sources of information section to include the fact that these were watched at school in the past**

Lines 390-392: It seems that this part describes the main findings (summary) of this study, so it may better to put it into the first part of discussion.

**Response: We have retained the current position, because we feel this sums up the findings, before moving into the discussion. We recognize that there are different approaches to where a short summary is placed in a manuscript, but given the rich qualitative nature of the findings we feel that this nicely summaries our main results – and hope that the reviewer will understand our choice.**

Reviewer #1: - The conclusions are directly derived from the results, and the authors have discussed the implications of the findings and recommended specific actions to improve the control of schistosomiasis in the studied communities.

- Limitations of the study should be clearly described**.**

**Response: We have now included the limitations, in a specific ‘Study limitations’ section in the discussion.**

Reviewer #2: - In the discussion section, the paragraph of lines 458-463: You mentioned need of improved training for teachers (line 461). I felt “teachers” suddenly appeared. Could you add more explanation or justification for this?

**Response: Thank you for highlighting this. We have now included teachers in the list of participants as a source of information (page 6 and Table 1) and clarified the point that some information from school children comes from the teachers.**

-	Could you please describe the limitation of the study?

**Response: As above. We now have a Study limitations’ section in the discussion.**

Reviewer #1: A more careful proofread is needed to correct some mistakes

**Response: We apologise for the errors – which we have endeavoured to correct.**

SPECIFIC COMMENTS

1. Study justification should be improved, including justification for choosing rapid ethnographic assessment approach for the study. This can be added to introduction, before the last paragraph.

Generally, “lacking rigor” is a main criticism of qualitative studies that utilized rapid data collection approaches; however, these approaches are useful when we need more information about a specific problem. Typically, the approach used was essential to explore why schistosomiasis burden continued high in the studied communities despite long-term MDA. Answers might be not possible through quantitative research. Introducing these approaches in introduction is needed. Statements in lines 145 – 148 can be used with more elaboration. Be specific with schistosomiasis type (i.e. intestinal schisto. caused by S. mansoni).

**Response: A paragraph has been added on page 5 in the introduction explaining why the rapid ethnographic assessment was used and the specific contribution it makes in terms of understanding local ways of understanding schistosomiasis and community suggestions for improvement.**

2. Likewise, usefulness of the rapid ethnographic assessment approach can be discussed in discussion section. Discuss how the findings by this approach complement findings of quantitative research and how the findings would be useful to adjust or improve the current control policies.

**Response: We have added the section on page 5, and an expanded description in the methods. We hope that this provides the background on the value of the approach. We also cite work which has detailed the method which we have published. We have also enhanced the discussion of our approach in the discussion, within the limitations section.**

3. Readers would be interested in reading description of environmental sources of infection based on observations and experience of living in the communities. What were the sources of infections? Just the lake! or small ponds also present? What about related agricultural activities and who were involved? Washing clothes or utensils! describe the situation and activities? What other occasions in these communities that may involve exposure to infection?

**Response: Thank you for suggesting this. Further detail has been added in the ‘Study area’ section in the methods regarding occupational and other forms of water contact.**

4. Rapid ethnographic assessment methods are not meant to produce quantitative data and statistical results. However, some information on the respondents or study activities and communities can be provided in a table, if available. For instance, a table that may include,

- Distribution of respondents from each community according to gender, age, community categories, and any other general characteristics.

- Differences between communities in terms of water sources, toilet facilities, etc.

- Acceptable/correct opinions, main misconceptions, etc.

**Response: As the reviewer has noted, ethnographic methods are not designed to produce quantitative data, and as a result we are extremely reluctant to extrapolate out from an explicitly exploratory design to make quantified or quantifiable claims. Also as this paper focuses on messages and it is not known exactly what type of messages are good or bad, helpful or a hinderance etc, we would not like to categories these findings. I agree that specific messages can be correct or incorrect, but we are not reporting on the actual messages themselves, but the way that people have perceived them and report passing them on, so we therefore remain reticent to include these details in a table.**

**Regarding the age, gender, community categories for the participants, as they were selected equally by age and gender these data do not add to the story and may turn participants into numbers rather than individuals.**

5. I also recommend adding a table or a box for the rapid ethnographic assessment approach of schistosomiasis as an ethnographic field guide for future studies in other endemic communities in Uganda or elsewhere. This will support the citation of this work.

**Response : Thank you for this suggestion which we are happy to provide and agree it enhances the work and reproducibility. We have added Table 1.**

6. Nothing is mentioned about *S. haematobium* and the classical sign hematuria. Is S*. haematobium* completely non-exist in these communities? If not, then why not included? Why messaging was only about defecation in water and not include urination? etc. Please clarify.

**Response: *S. haematobium* is not present at all in these or nearby communities and thus the focus of the paper is on *S. mansoni*. We have made this clearer in the first paragraph of the paper and in the methods. Thank you for highlighting this.**

7. Limitations of the study should be clearly described at the end of discussion.

**Response: This has been included in the discussion**

8. A map of study area can be added to show the location of the district and studied communities with water bodies and streams.

**Response: A map of Uganda, Mayuge District and the three study communities has now been added.**

9. I suggest indicating the approach in the title “Rapid ethnographic assessment”. I do believe that this approach is important to explore complex social issues as well as barriers of NTDs control in minor and hard-to-reach communities. The title may encourage other authors elsewhere to adopt similar approaches.

**Response: Thank you. We have added the data collection method in title**

10. Lines 78-87: “Parasite eggs …….. diseases [2].” This general information can be removed.

**Response: We have shortened that section, and used that text to stress the focus on S. mansoni.**

11. Introduction, 2nd paragraph: a nice paragraph but it can be shortened.

**Response: Thank you. We have condensed.**

12. Line 33: continued exposure!

**Response: Corrected**

13. Line 109: correct “inpractical” to impractical.

**Response: Thank you – the whole section has been reworked so this error has been removed.**

14. Line 121: of these! Rephrase.

**Response: Thank you. We have corrected.**

15. Line 123: change “uncontaminated water” to “improved water”.

**Response: This has been changed**

16. Line 151: correct appraisal to appraisal. What about naming it as rapid ethnographic assessment?

**Response: This has been renamed**

17. Line 152: “Two social science trained research assistants”. Add more information: Were the assistants from the local communities? Were the assistants fluent in the local language? Were the assistants had prior experience in the field of schistosomiasis/NTDs/parasitology?

**Response: Additional information has been inserted about the research assistants. They were well trained social scientists with experience working on various studies and fluent in the local language.**

18. Lines 240-242: this can be removed.

**Response: This has been removed.**

19. Line 242: what were the suggestions made by some community members? Nothing mentioned about this.-

**Response: This was misplaced, all the findings are from the community members. We have reworked this sentence.**

20. Line 338: what did they mean by Illuminati?

**Response: This refers to a satanic group who are a form of cult according to the community members. An explanation has now been added.**

21. Line 424:” praziquantel drugs may affect fertility”! you may cite respondents’ statements.

**Respondent: Given this is in the discussion, we have not included information from the respondents, but we have expanded on this observation and added a citation in the third paragraph of the discussion.**

22. As the study covered *S. mansoni* only, intestinal schistosomiasis should be mentioned clearly in the text, at least in the introduction and discussion.

**Response: We have reworked the first paragraph to make the focus on S. mansoni and intestinal schistosomiasis clear.**

23. A more careful proofread is needed to correct some mistakes.

**Response: Apologies. This has been done.**

Editor comments on resubmission:

1) Please ensure that the CRediT author contributions listed for every co-author are completed accurately and in full.

At this stage "all Authors" require contributions. Please ensure that the full contributions of each author are acknowledged in the ""Add/Edit/Remove Authors"" section of our submission form.

**We have updated the author CRediT contributions**

2) Please provide separate figure files in .tif or .eps format. You do not need to remove any figures embedded in your manuscript file, so long as you have provided separate figure files. Please ensure that all files are under our size limit of 20MB.

**We have now updated the map to include a Natural Earth basemap to comply with PLoS publications and also provided it in the correct format.**

3) Thank you for including your ethics statement: "The parents or legal guardians of the children were asked for consent before their children were included in the discussion groups. The children were asked for their assent". Please state whether written/verbal consent was obtained from the parents or legal guardians of the children.

**We have confirmed in the manuscript that we obtained written consent from the parents/legal guardians and verbal assent from the children.**

4) We have noticed that you have uploaded supporting information but you have not included a list of legends. Please add a full list of legends for all supporting information files (including figures, table and data files) after the references list.

**We have now included legends for the supporting information in the text.**

5) Please ensure that the funders and grant numbers match between the Financial Disclosure field and the Funding Information tab in your submission form. Note that the funders must be provided in the same order in both places as well. Currently, the order of the funders and grants differs in both places.

**We have amended the order of the funders in the funding disclosure section to match the manuscript and corrected the ERC funding.**

6) In the online submission form, you indicated that your data will be submitted to a repository upon publication. We strongly recommend all authors deposit their data before acceptance, as the process can be lengthy and hold up publication timelines. Please note that, though access restrictions are acceptable now, your entire data will need to be made freely accessible if your manuscript is accepted for publication. This policy applies to all data except where public deposition would breach compliance with the protocol approved by your research ethics board. If you are unable to adhere to our open data policy, please kindly revise your statement to explain your reasoning and we will seek the editor's input on an exemption. Please be assured that, once you have provided your new statement, the assessment of your exemption will not hold up the peer review process.

**We have now made the anonymised raw data, extracted after coding from the full transcripts, available as Supporting Information 3. We cannot include the raw transcripts due to limitations with anonymity and how our informed consent was obtained, but we hope that this will suffice.**

**Many thanks for your excellent suggestions and we hope you agree that the manuscript is now greatly improved and we look forwards to hearing from you.**

**Kind regards**
